# ASGCL: Adaptive Sparse Mapping-based graph contrastive learning network for cancer drug response prediction

**DOI:** 10.1371/journal.pcbi.1012748

**Published:** 2025-01-30

**Authors:** Yunyun Dong, Yuanrong Zhang, Yuhua Qian, Yiming Zhao, Ziting Yang, Xiufang Feng

**Affiliations:** 1 School of Software, Taiyuan University of Technology, Taiyuan, China; 2 Institute of Big Data Science and Industry, Shanxi University, Taiyuan, China; 3 School of Computer and Information Technology, Shanxi University, Taiyuan, China; Rhodes University, SOUTH AFRICA

## Abstract

Personalized cancer drug treatment is emerging as a frontier issue in modern medical research. Considering the genomic differences among cancer patients, determining the most effective drug treatment plan is a complex and crucial task. In response to these challenges, this study introduces the Adaptive Sparse Graph Contrastive Learning Network (ASGCL), an innovative approach to unraveling latent interactions in the complex context of cancer cell lines and drugs. The core of ASGCL is the GraphMorpher module, an innovative component that enhances the input graph structure via strategic node attribute masking and topological pruning. By contrasting the augmented graph with the original input, the model delineates distinct positive and negative sample sets at both node and graph levels. This dual-level contrastive approach significantly amplifies the model’s discriminatory prowess in identifying nuanced drug responses. Leveraging a synergistic combination of supervised and contrastive loss, ASGCL accomplishes end-to-end learning of feature representations, substantially outperforming existing methodologies. Comprehensive ablation studies underscore the efficacy of each component, corroborating the model’s robustness. Experimental evaluations further illuminate ASGCL’s proficiency in predicting drug responses, offering a potent tool for guiding clinical decision-making in cancer therapy.

## Author summary

Cancer is a globally challenging disease characterized by significant heterogeneity, predicting anti-cancer drug sensitivity plays critical roles in drug development and developing personalized drug treatment plans for cancer. Some computational methods have been proposed to address interactions between multiple cell lines and drugs, and provide promising results. However, the existing methods fail to fully consider the potential complex non-linear relationships and struggle to capture long-range dependencies in the underlying graph. To overcome above questions, a novel computational method named ASGCL model is proposed to amalgamate the characteristics and documented reactions of cancer cell lines and drugs. ASGCL constructs graph augmentation module GraphMorpher, and generates enhanced graphs, preserving key structures and attributes while sparsifying secondary features and topologies in each iteration. The experimental results show that ASGCL is able to effectively integrate data of cancer cell lines and drugs with existing response activities.

## Introduction

Cancer’s global challenge lies in its vast heterogeneity. Even within the same cancer type, drug responses vary due to genetic differences [1,2,3], emphasizing the urgency of personalized cancer treatments, a core aim in precision medicine. In drug development, predicting anti-cancer drug sensitivity has become a central research focus, involving genomics, transcriptomics, proteomics, and related disciplines[4,5], [6]. Traditional prediction methods, based on statistical matrix decomposition [7], rely on data’s statistical features, overlooking uncertainties and incompleteness. This limitation makes it challenging to address interactions between multiple cell lines and drugs, leading to inaccuracies and misjudgments in predictions.

With the development of artificial intelligence, machine learning and deep learning methods for predicting drug resistance are emerging. For instance, classic machine learning algorithms like logistic regression [8], support vector machines [9], and random forests [10] have shown significant effectiveness in drug resistance prediction. These methods are primarily based on single-omics data such as genetic mutations or gene expression. However, integrating multi-omics data allows for the extraction of more advanced correlations, thereby enhancing their predictive capabilities. For instance, NetLapRLS [11] utilizes the Laplacian regularized least squares method, combining known drug-target interactions, chemical structures, and genomic sequence data for drug resistance prediction.

With the advancement of genomics, the application of novel genomic data [4,12,13] in predicting drug resistance [14,15] is increasingly prevalent. For instance, the method proposed by Su et al. [16] transforms cell line gene expression and copy number variations into high-dimensional feature vectors to predict drug responses in corresponding cell lines. However, most current machine learning algorithms model the correlation between cell line features and drug features linearly, failing to fully consider the potential complex non-linear relationships.

Another approach to predicting drug resistance focuses on using deep learning models to extract features of cell lines and drugs and input them into classifiers to predict the cells’ responses to drugs [15,16,17,18,19,20,21]. Various deep neural networks, like multilayer perceptrons [22,23], convolutional neural networks [21,22,23,24], and recurrent neural networks [25], have been used to learn potential representations of cancer cell lines and drugs. Hossein et al. [15] encode gene expression, somatic mutations, and copy number variations of cell lines, creating feature representations of these lines. However, most feature-based drug resistance prediction methods overlook the associations between cell lines or drugs. To address this, Zhang et al. [19] established a dual similarity network based on cancer cell line gene expression and drug chemical structures for predicting drug resistance. Turki et al. [26] introduced a link-filtering algorithm based on the cancer cell line network, combined with a linear regression model for the prediction task.

Recent research in bioinformatics shows that graph structures excel in predicting drug resistance, current methods primarily use graph structures for modeling nonlinear structural features of cell lines and molecular structures of drugs. For instance, DeepCDR [21] is a hybrid prediction model based on GCN, integrating data expression. HNMDRP [27] constructs a heterogeneous network of cell line-drug targets and employs a flow-based prediction algorithm. In general, these graph-based methods extract cell line features from gene expression, somatic mutations, and copy number variations, and combine them with drug FingerPrints and SMILES molecular structures to create molecular graphs for extracting drug features [20]. However, due to the increasing layers in graph neural networks lead to over-smoothing issues, making the final feature representations less distinct and affecting prediction accuracy.

Given the limited availability of detailed annotated data on cancer cell, self-supervised learning approach focuses on automatically extracting features from data. Contrastive learning [28,29,30] aims to enhance sample processing by dividing them into positive and negative sets, striving to maximize the similarity within positive sets and minimize it within negative sets. [30,31,32]. For example, GraphCDR [33] creates contrastive learning tasks for cancer cell line drug resistance reactions, thereby enhancing the model’s generalization capability and learning richer feature representations.

In our study, we proposed an Adaptive Sparse Mapping based Graph Contrastive Learning Network (ASGCL) for predicting cancer drug response. Extracting the original features of cell lines and drugs through nonlinear subspaces, mapping them into heterogeneous graphs through cell line drug interactions, and then proposing the GraphMorpher module to perform adaptive graph sparsity on heterogeneous graphs. Finally, a contrastive learning task is designed on multiple graph structures to enhance feature discrimination, and a graph encoder is used to learn potential features of cell lines and drugs from sensitive graphs for supervised prediction tasks. The contributions of this paper are as follows:

⚫ Within the graph contrastive learning framework, the ASGCL model amalgamates the characteristics and documented reactions of cancer cell lines and drugs. By integrating multi-omics information, the predictive performance of cancer drug responses has been significantly improved.
⚫ This study innovatively developed the adaptive graph augmentation module GraphMorpher, which performs masking operations on nodes and pruning operations on links. In each iteration, the module adaptively generates enhanced graphs, preserving key structures and attributes while sparsifying secondary features and topologies.
⚫ Additionally, by implementing contrastive learning tasks, this study greatly improved the model’s generalization capability, defining positive and negative samples at the node and graph levels and setting multi-level learning objectives, further enhancing the model’s discrimination ability.

## Related work

### Similarity matrices

Numerous statistical-based methods have been proposed for predicting drug resistance, including techniques such as matrix decomposition. For instance, SRMF [7] is a matrix decomposition algorithm that analyzes cell line and drug characteristics by decomposing known cell line-drug relation matrices and predicts drug responses. DTINet [34] learns low-dimensional representations of drugs and proteins from multiple networks and employs inductive matrix completion techniques to predict drug-target interactions. Zhang et al. [27] constructed a heterogeneous network model by calculating the Pearson correlation coefficients between cell line genomic profiles, drug chemical structures, and target genes. However, these methods primarily focus on identifying drug-cell line interactions through linear combinations of latent features. In reality, interactions between drugs and individual cell lines may exhibit significant non-linearity.

Matrix decomposition-based methods [35,36] typically involve multiplying decomposition factors to reconstruct known drug-response relationships, that these factors are constrained by side information from cancer cell lines and drugs [37,38], potentially impacting the task of predicting drug resistance.

### Graph Neural Networks (GNN)

With the capability to learn complex functions and high-dimensional representations continues to improve,particularly, models based on GNN [39,40] are notably significant in exploring relationships between drugs and cell lines due to their effective use of graph topology to aggregate node features.

For instance, GraphDRP [20] obtains the embedded representation of drugs through GCN, while using binary vectors of genomic mutations to acquire the embedded representation of cell lines. NIHGCN [41] constructs a cell line-drug heterogeneous network, using cell line gene expression and drug fingerprints as node features. Wei et al. [42] proposed an end-to-end algorithm named MOFGCN that based on multi-omics integration and GCN to predict drug responses in cell lines.

The above research methods based on graph structures have achieved many academic results. Although most methods consider the relationships between drugs and cell lines when constructing model structures, many methods for modeling cell lines and drug structures do not take into account the global features of graph structures, blindly using multi-layer GCNs, gradually covering the features with the features of message transmission mechanisms, resulting in the convergence of features between nodes with message transmission.

### Contrastive learning

Although existing methods have achieved significant results, most models rely on label-supervised training, which is limited by the size of datasets and the scarcity of task-specific labels in graph-structured datasets.

In response to this challenge, some previous research has introduced contrastive learning in drug resistance tasks. For instance, Yao et al. [43] developed the SHGCL-DTI framework by incorporating a contrastive learning module in semi-supervised prediction tasks. Graph contrastive learning provides a new framework for learning feature representations in graph structures where labeled data is limited or absent. For example, Liu et al. [44] selected k-nearest neighbor nodes as positive samples for each node and selected negative samples from the remaining nodes in the graph.

Furthermore, existing studies [33,34,43,44,45,46,47,48] have not yet fully explored the rich semantic information and complex interactions between data in heterogeneous graphs. Wei et al. [49] proposed a contrastive learning model, which integrating contrastive learning strategies into graph collaborative filtering. Gao et al. [50] introduced a graph joint contrastive learning model, using network topology for learning embeddings. Most of the above studies focus on generating similar positive samples within graph structures to learn similar features, but they often overlook the importance of negative samples.

## Results

### Dataset

We conducted experimental tests of our model on two datasets: GDSC and CCLE. This study utilized the GDSC dataset to determine the sensitivity of cell lines to drugs, defining cell line-drug pairs as sensitive if their IC50 values exceeded the threshold specified in the threshold table. The GDSC database has 990 cancer cell lines and 265 drugs, with 20,851 sensitive samples and 156,512 resistant samples. For the CCLE dataset, the log10(IC50) values of all cell lines were normalized to a zero mean and unit variance. If the Z-score normalized log10 (IC50) value is less than -0.8, the cell line is defined as sensitive to the drug. Otherwise, the cell line is considered to be resistant to the drug. Ultimately, the CCLE dataset comprised 1,696 sensitive samples and 8,768 resistant samples. We extracted gene expression, copy number variations, and somatic mutation histological features of the respective cell lines from the GDSC. Additionally, we removed from PubChem cell lines lacking any histological data and drugs with identical compound IDs (CIDs). After processing, we obtained the gene expression, copy number variations, and somatic mutation histological features of 962 cell lines, along with their responses to 228 drugs.

### Experimental details

We utilize the GPU of RTX Nvidia 4090, conducting training and testing based on the PyTorch framework. The dataset is divided into training and test sets using five-fold cross-validation, and the model parameters are updated using the Adam optimizer. In the experiments, the learning rate is set to 5e-4; the number of hidden layers is configured as (962,224), with the hidden layers in the graph encoder set to (224,24). The settings of other parameters will be substantiated in subsequent experiments.

In our model, a nonlinear subspace encoder is constructed, and a selection is made among Graph Neural Network (GNN), Graph Convolutional Network (GCN) and Graph Attention Network (GAT). Ultimately, we chose GCN (k = 1) as the nonlinear subspace encoder. The detailed comparison process is provided in the ablation study section.

### Evaluation criteria

This paper evaluates the performance of the model using the Area Under the Receiver Operating Characteristic curve (AUC), Average Precision (AP), Accuracy (ACC), F1 Score, and Matthews Correlation Coefficient (MCC). Mathematically, these metrics are defined as follows:

$$Precision=\frac{TP}{TP+FP}$$ (1)

$$ACC=\frac{TP}{TP+TN}$$ (2)

$$Recall=\frac{TP}{TP+FN}$$ (3)

$$F1-score=\frac{2TP}{2TP+FP+FN}$$ (4)

$$MCC=\frac{TP\times TN-FP\times FN}{\sqrt{\left(TP+FP)(TP+FN)(TN+FP)(TN+FN\right)}}$$ (5)

where true Positives (TP), True Negatives (TN), False Negatives (FN), and False Positives (FP) are the four categories of the confusion matrix, calculated by comparing predicted values with actual response values. The F1 Score is defined as the harmonic mean of precision and recall. The MCC produces a high score only when the prediction yields good results across all four categories of the confusion matrix (TP, TN, FN, and FP).

### Loss function

In this study, contrastive loss and supervised loss are combined in an end-to-end manner to learn feature representations. The extent of loss for supervised tasks is determined using the cross-entropy loss function, which can be represented as:

$${Loss}_{sup}=-\frac{1}{\left|S\right|}\sum \left(P_{lab}\log\tilde{P}+(1-P_{lab})\log\tilde{P}\right)$$ (6)

where S represents the training set of the same batch, *P*_*lab*_ the actual label values between nodes, and $\tilde{P}$ the predicted values between nodes.

To simultaneously accomplish the task of drug resistance prediction and contrastive learning, the losses from contrastive learning and supervised learning are combined, optimizing the following loss function:

$$Loss=\alpha \cdot {Loss}_{sup}+\beta {Loss}_{nod}+\gamma {Loss}_{gra}$$ (7)

where *α*,*β* and *γ* are hyperparameters representing the contributions of different loss functions, with *α*+*β*+*γ* = 1. In our study, we set the hyperparameter *α* to 0.4 and the hyperparameters *β* and *γ* to 0.4 and 0.2, respectively, maintaining a 2:1 ratio.

### Experimental results

To verify the effectiveness of our proposed ASGCL model, multi-method comparative experiments were conducted in this section, along with quantitative and qualitative assessments of these methods. The methods used in the comparative experiments include DeepCDR, DeepDSC, MOFGCN, GraphCDR, and TSGCNN. All comparison methods are based on the same datasets and evaluation metrics, and the default or optimal parameter settings for each model were adopted. All benchmark models utilized the same input features and preprocessing strategies as ASGCL. Stratified 5-fold cross-validation was used to assess model performance. Specifically, the samples in the dataset were divided into five equal parts, each part taking turns as the test set, with the remainder serving as the training set. In each fold, the performance of different models was evaluated using the average of various metrics.

The experimental results, as shown in Table 1, indicate that the ASGCL model achieved the highest performance on both the GDSC and CCLE datasets compared to baseline models. Compared to other models, ASGCL showed superior performance with the highest AUC (96.23%), AP (97.73%), ACC (90.32%), F1 Score (89.66%), and MCC (81.33%), indicating a good match between IC50 values and predicted values. The main reason is that the model proposed in this paper obtained reliable feature representations of cell lines and drugs through contrastive learning on graph structures. In other models, the feature representations were randomly initialized, neglecting the rich biomedical information related to cell lines and drugs. These results demonstrate that the ASGCL model is more effective in extracting additional drug potential data, and the learned embeddings are more representative.

**Table 1:** Performance comparison of different algorithms based on five indicators.

Dataset	Algorithm	AUC	AP	ACC	F1_Score	MCC
**GDSC**	DeepCDR	0.8009±1×10^−2^	0.8091±1×10^−2^	0.7748±1×10^−2^	0.8063±1×10^−3^	0.5812±1×10^−2^
	DeepDSC	0.8174±1×10^−2^	0.8327±1×10^−3^	0.7901±1×10^−2^	0.8154±1×10^−3^	0.6073±1×10^−2^
	MOFGCN	0.8785±1×10^−5^	0.8866±1×10^−3^	0.8446±1×10^−3^	0.8576±1×10^−3^	0.7069±1×10^−2^
	GraphCDR	0.8076±1×10^−3^	0.8144±1×10^−3^	0.7819±1×10^−3^	0.8107±1×10^−3^	0.5946±1×10^−2^
	TSGCNN	0.9156±1×10^−3^	0.9182±1×10^−3^	0.8833±1×10^−3^	0.8906±1×10^−3^	0.7779±1×10^−2^
	**Ours**	**0.9623±1×10** ^**−3**^	**0.9773±1×10** ^**−3**^	**0.9032±1×10** ^**−3**^	**0.8966±1×10** ^**−3**^	**0.8133±1×10** ^**−3**^
**CCLE**	DeepCDR	0.8322±1×10^−3^	0.8400±1×10^−3^	0.8047±1×10^−3^	0.8246±1×10^−3^	0.6337±1×10^−2^
	DeepDSC	0.8497±1×10^−3^	0.8649±1×10^−3^	0.8239±1×10^−3^	0.8373±1×10^−3^	0.6660±1×10^−2^
	MOFGCN	0.8442±1×10^−3^	0.8563±1×10^−3^	0.8191±1×10^−3^	0.8344±1×10^−3^	0.6583±1×10^−3^
	GraphCDR	0.8438±1×10^−3^	0.8520±1×10^−3^	0.8160±1×10^−3^	0.8317±1×10^−3^	0.6526±1×10^−2^
	TSGCNN	0.8823±1×10^−3^	0.8908±1×10^−3^	0.8471±1×10^−3^	0.8585±1×10^−3^	0.7108±1×10^−2^
	**Ours**	**0.8925±1×10** ^**−3**^	**0.9564±1×10** ^**−3**^	**0.8443±1×10** ^**−3**^	**0.8568±1×10** ^**−3**^	**0.7963±1×10** ^**−3**^

### Classification task results

**Fig 1:**
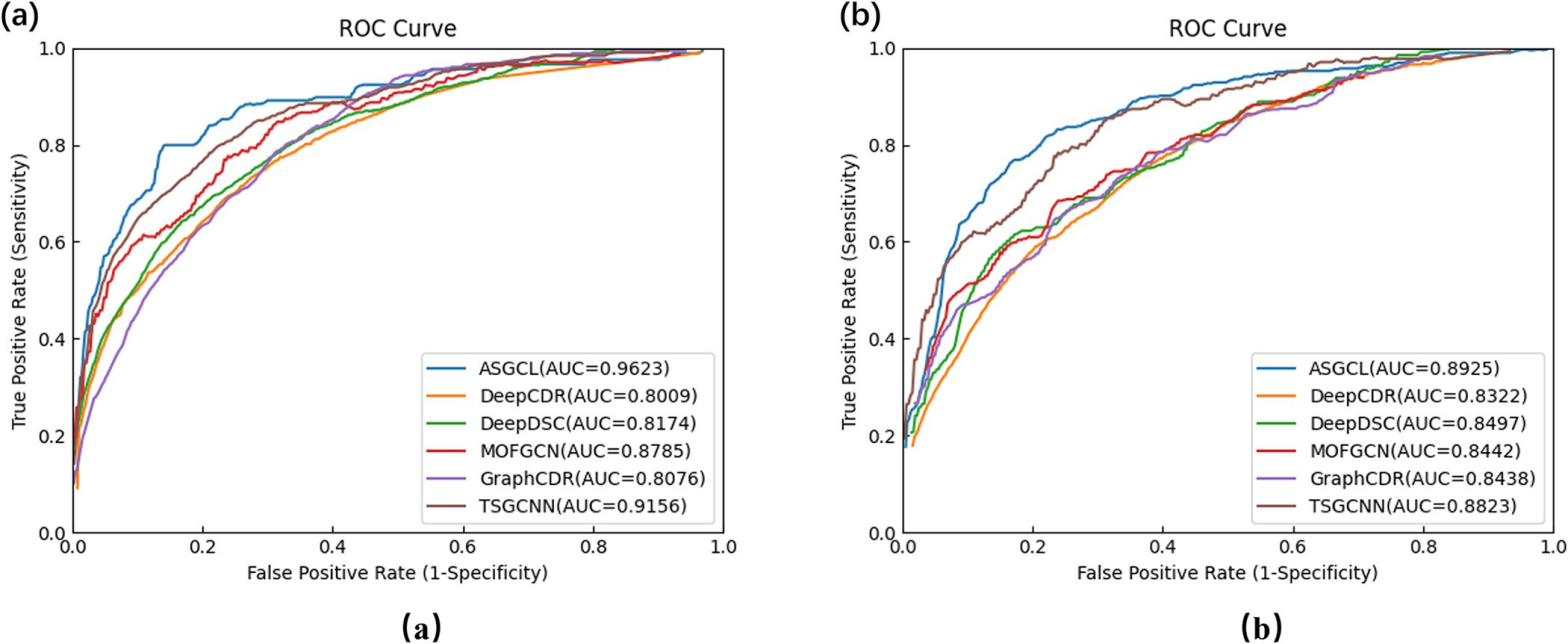
ROC curves of ASGCL model and five comparison models. (a) ROC curves on GDSC dataset (b) ROC curves on CCLE dataset.

To further assess the performance of prediction models on the GDSC and CCLE datasets, this study conducted an independent validation, testing the performance of the proposed ASGCL model and comparison models in drug response classification tasks, using ROC curves as the evaluation metric. As hyperparameter tuning and model training are independent of the separate test set, the separate test set can better measure the ASGCL model’s generalization ability on unknown data. As shown in Fig 1, Fig 1A depicts the ROC curve of the ASGCL model on the GDSC dataset, where the horizontal axis represents the false positive rate and the vertical axis represents the true positive rate; Fig 1B shows the ROC curve of the ASGCL model on the CCLE dataset. According to the figures, the ASGCL model scored 96.23% in AUC on the GDSC dataset and 89.25% in AUC on the CCLE dataset, both higher than the baseline in tests on the two datasets. The independent test results indicate that the ASGCL model has a high generalization ability.

### Predicting task results

**Fig 2:**
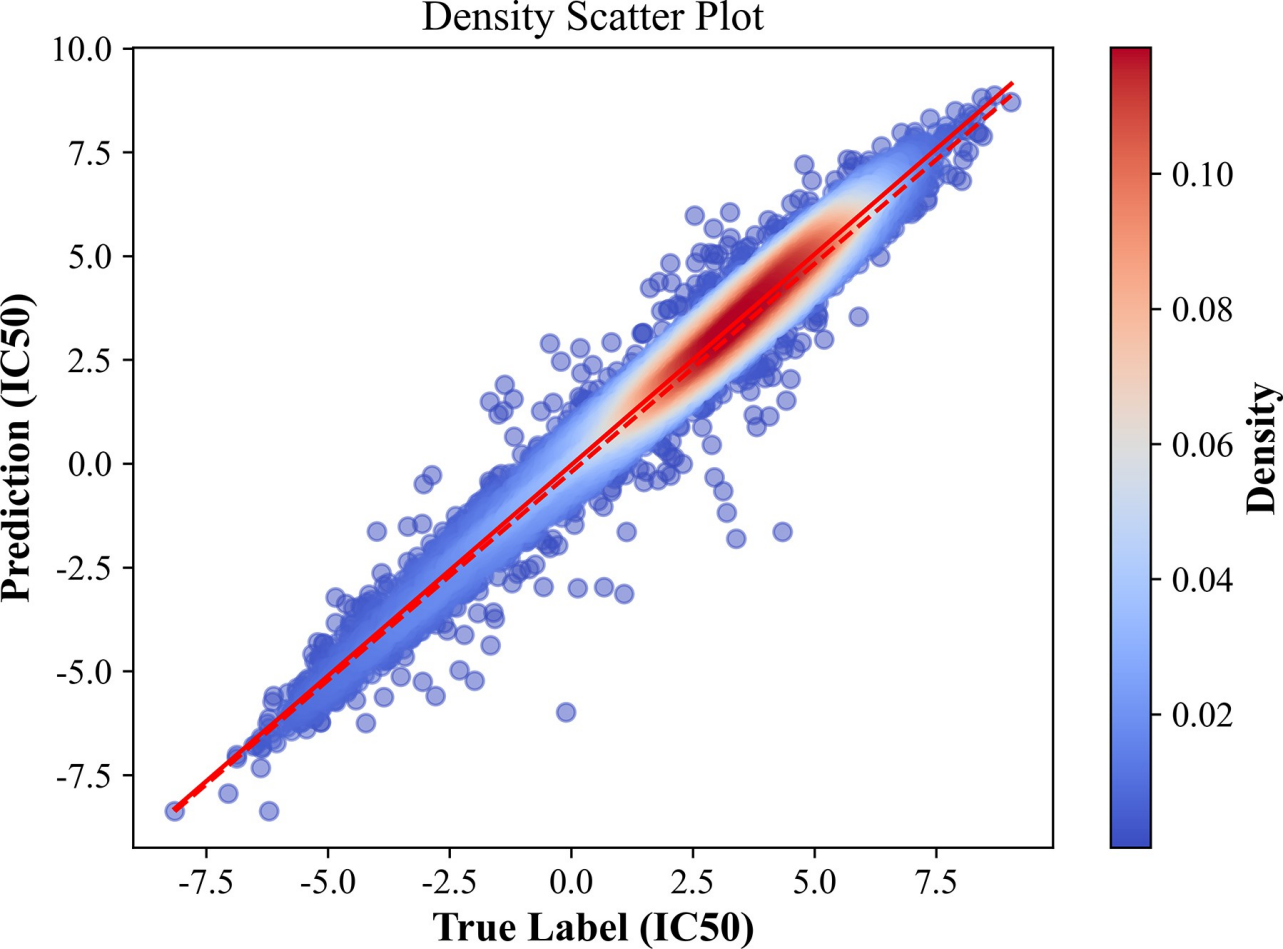
Scatter plot of predicting drug response (IC50 value) in GDSC using ASGCL model.

Fig 2 visualizes the performance of the ASGCL model in the drug response regression task. The horizontal axis represents the actual IC50 values, and the vertical axis represents the predicted IC50 values, with the color gradient from light to dark indicating increasing density. The solid red line represents the prediction results of linear fitting based on the data; the dashed red line represents the ideal scenario where the predicted results completely match the actual results. Visualization through scatter plots of the distribution of actual versus predicted values shows a good correlation between actual and predicted responses, demonstrating that the proposed ASGCL model exhibits good predictive performance for a large number of random samples.

### Prediction of response in unknown drug cell lines

**Fig 3:**
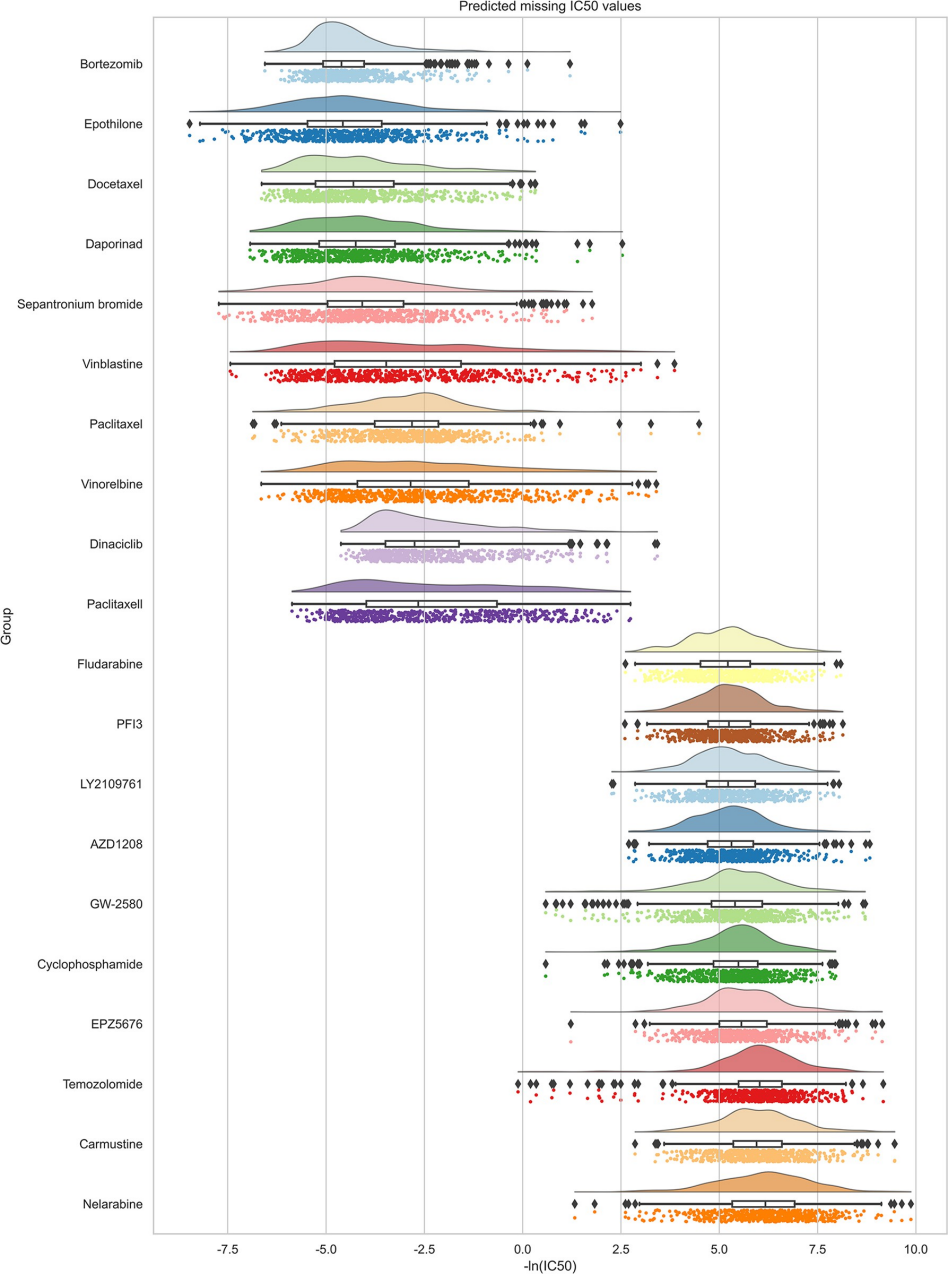
Predict the IC50 values of unknown cell line drug reactions grouped by drugs. Drugs are classified based on the median predicted IC50 values of all missing cell lines, and the top 10 drugs with the highest median IC50 have the worst efficacy; The last 10 drugs with the lowest IC50 median may be the most effective.

We established the ASGCL model using all known cell line-drug reactions and predicted reactions for unknown cell line-drug pairs, as shown in Fig 3.

We applied the ASGCL model to predict unknown cell line-drug reactions in the GDSC database. ASGCL was trained on all known interactions between 962 cell lines and 228 drugs, then predicted missing IC50 values in the GDSC database. Predictions were grouped by drug and then sorted by the median of the predicted IC50 values. The raincloud plots of the top 10 drugs with the highest and lowest median IC50 values are shown in Fig 3. After testing each drug’s distribution, more than 17 out of 20 selected drugs showed no significant difference between their IC50 values and the distribution of predicted unknown IC50 values. The results indicate that the predicted missing IC50 values match the distribution of actual measurements, suggesting that our model can effectively distinguish between sensitive and resistant drugs. Since these unknown DCPs lack real values, the results were validated based on relevant literature. Specifically, Bortezomib [51] was predicted to be the most effective drug, consistent with previous studies that suggest Bortezomib can inhibit cancer by interacting with various cancer cell lines.

### Ablation

This paper conducted ablation experiments on the GDSC dataset for the nonlinear subspace, GraphMorpher module, and graph encoder used in the contrastive learning stage of the ASGCL model, studying the effectiveness of these modules by replacing or eliminating each of them.

### Evaluation of the nonlinear subspace

In this study, we used a nonlinear subspace approach to merge linear and nonlinear features. Although existing linear methods perform well in extracting features of cell lines and drugs, relying solely on linear feature extraction overlooks the topological structure features in cell lines and drugs. Therefore, a nonlinear subspace was introduced to merge linear and nonlinear features, extracting more effective original features.

**Fig 4:**
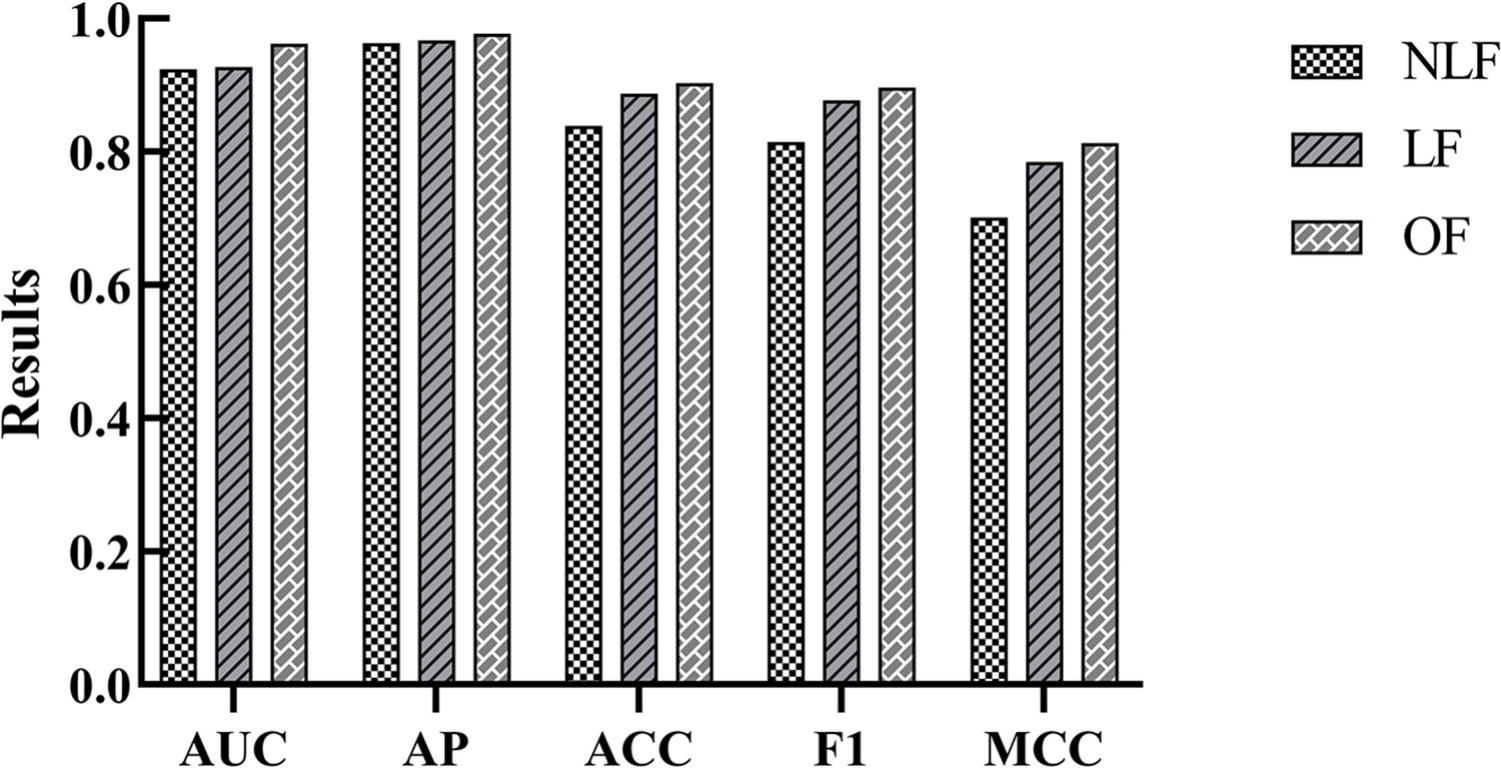
Research on ablation of nonlinear subspaces.

To evaluate the effect of using a nonlinear subspace in the model, a series of ablation experiments were conducted (Table 2). Comparisons were made between using only linear features, only nonlinear features, and combining both. Fig 4 shows the comparison between the performance of the model using a nonlinear subspace and models that only extract linear or nonlinear features.

**Table 2:** Ablation experiments using different contrasting methods.

Original Graph	Resistance Graph	Node Mask	Link Pruning	AUC	AP	F1_Score
**✔**				0.9045	0.9448	0.8351
**✔**	**✔**			0.9384	0.9712	0.8519
**✔**	**✔**	**✔**		0.9315	0.9615	0.8727
**✔**	**✔**		**✔**	0.9240	0.9626	0.8148
**✔**	**✔**	**✔**	**✔**	**0.9623**	**0.9773**	**0.8966**

The results show that the performance of the model using a nonlinear subspace is significantly better than models that only extract linear or nonlinear features. This indicates that the subsequent contrastive learning process largely depends on effective initial features. By introducing a nonlinear subspace, complex relationships between cell lines and drugs can be better captured, thereby improving the performance of the model. In summary, the method of using a nonlinear subspace in this study can effectively merge linear and nonlinear features and extract more informative original features.

### Evaluation of contrastive learning

This study introduces contrastive learning to enhance the quality of feature extraction. Although supervised learning has been effective, the addition of contrastive learning enables the model to more effectively identify and extract key information in the graph, thus obtaining more distinctive feature representations. To verify the role of contrastive learning in the ASGCL model, this paper conducted ablation experiments to demonstrate the effectiveness of the proposed method in the following five parts: (1) Directly performing drug resistance prediction tasks on the features of the original graph, without contrastive learning; (2) Only conducting contrastive learning on sensitive and resistant graphs, without learning nodes and topological structures; (3) Only performing node masking operations on the input graph structure; (4) Only performing link pruning operations on the input graph structure; (5) Performing node masking and link pruning operations separately on the input sensitive and resistant graphs.

### Evaluation of the graph encoder

**Fig 5:**
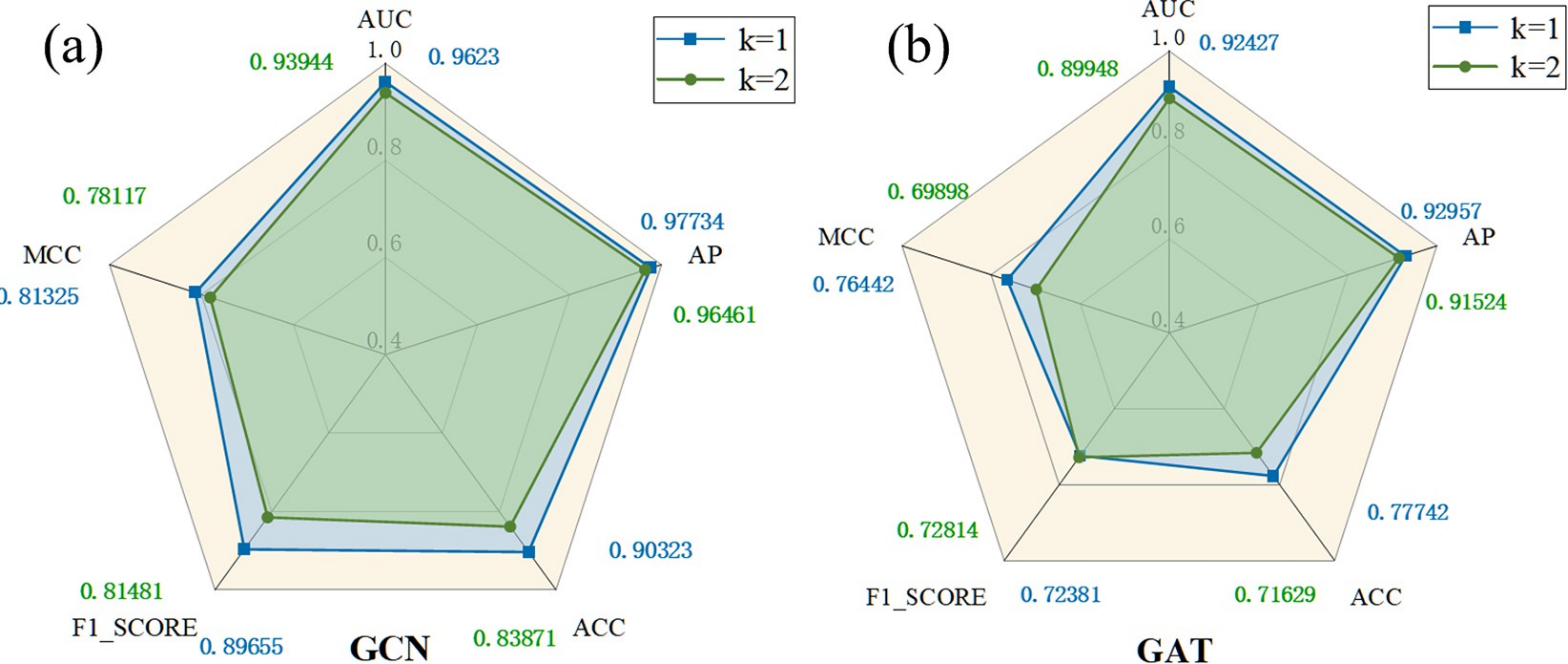
Comparative experiments on different graph encoders in different neighborhoods. (a) Experimental results of GCN on different neighborhood layers (b) Experimental results of GAT on different neighborhood layers

In this study, a graph encoder was used to encode the node attributes and their topological structure within the graph structure. During the model construction process, various commonly used encoders were considered, including Graph Convolutional Network (GCN) and Graph Attention Network (GAT). This paper investigated the impact of different encoders on the overall performance of the model through ablation experiments, as well as the effect of setting different convolutional layers in the graph encoder, with comparative results detailed in Fig 5. The experimental results showed that using GCN as the graph encoder with K = 1 yielded better overall effects than GAT, with an increase of 3.81% in AUC, and other performance metrics such as AP and F1 Score improved by 4.78% and 17.27% respectively. This result indicates that GCN has a significant advantage in encoding graph structures in this study. This superiority might be attributed to GCN’s ability to effectively capture key structural features of the graph while maintaining a simple structure. In contrast, the performance decline of GAT could be due to overfitting, as the specificity of cell line and drug data may not suit the complex attention mechanism.

The impact of the number of convolutional layers K on the model’s performance, as shown in Fig 5, indicates a decline when K increases from 1 to 2. This suggests that while a higher number of neighborhood layers may provide more valuable information, the specificity of the cell line and drug data in this study means that too high a value of K could introduce excessive noise or irrelevant information. Furthermore, the issue of over-smoothing could arise, leading to generated feature representations lacking distinctiveness, thereby negatively impacting model performance.

### Parameter experiments

In this section, we study the impact of several key parameters on the model. While assessing any parameter, default values are set for the others. The following parameter studies are conducted on the GDSC dataset.

### The roles of node masking probability $\rho^{N}$ and link pruning probability *ρ*^*ℒ*^ in GraphMorpher

**Fig 6:**
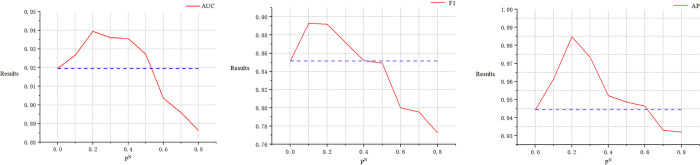
The impact of the parameter. $\rho^{N}$ on the ASGCL model’s performance is demonstrated, where the blue dotted line represents the baseline result of the ASGCL model on the GDSC dataset when the parameter *ρ*^ℒ^ is not introduced.

**Fig 7:**
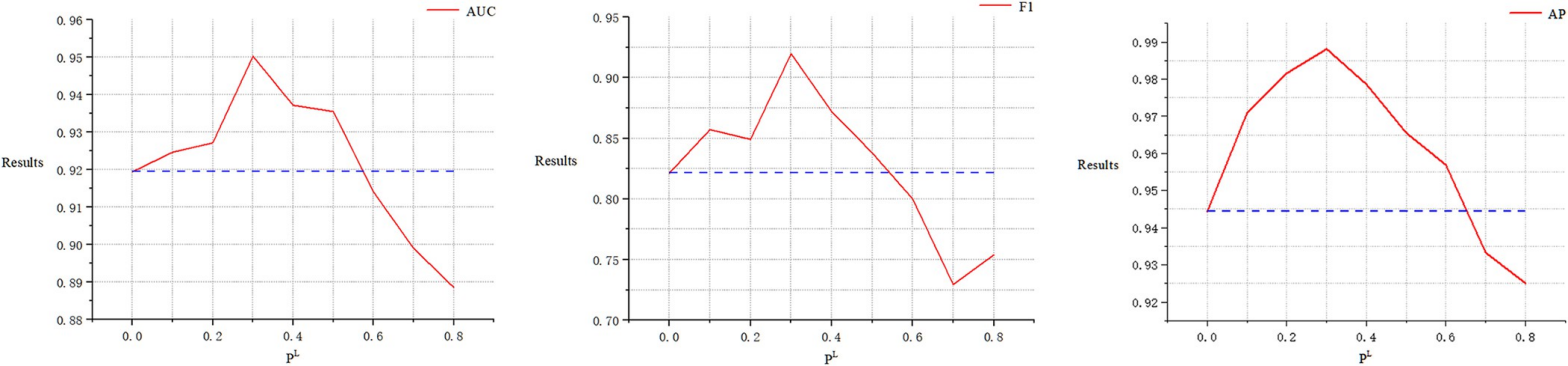
The impact of the parameter on *ρ*^ℒ^. The ASGCL model’s performance is demonstrated, where the blue dotted line represents the baseline result of the ASGCL model on the GDSC dataset when the parameter $\rho^{N}$ is not introduced.

Figs 6 and 7 analyze the impact of two different hyperparameters on segmentation performance. In the proposed GraphMorpher, $\rho^{N}$ represents the probability of node attributes in the graph structure being masked, and *ρ*^ℒ^ represents the probability of the topological structure being pruned. To study the impact of these two probabilities on experimental results, comparative experiments on these parameters were conducted in the dataset. The values of the two parameters were incrementally increased from 0.1 to 0.8 for experimentation, with a step size of 0.1. When experimenting with any one parameter, the other parameter was set to 0 to ensure that the remaining parameters did not affect the experiment. The results indicate that as the parameters $\rho^{N}$ and *ρ*^ℒ^ gradually increased, the effectiveness of the experiment first improved and then declined. Specifically, the best experimental effect was achieved when the node masking probability $\rho^{N}$ was at 0.2 and the topology pruning probability *ρ*^ℒ^ was at 0.3. As shown in the figure, the model’s effectiveness decreased as the values of $\rho^{N}$ and *ρ*^ℒ^ exceeded certain thresholds, likely due to the excessive probability of damaging nodes and links, causing the graph’s underlying semantic information and topological structure to suffer devastating damage, leading to feature loss. Based on the experimental results, we set the parameters $\rho^{N}$ and *ρ*^ℒ^ to 0.2 and 0.3 respectively in the experiments, and set the truncation probabilities $T_{N}$ and $T_{L}$ to 0.3 and 0.4, respectively, to prevent excessive probability of graph structure sparsification, ensuring the graph structure is not severely damaged.

### The combined role of node masking probability $\rho^{N}$ and link pruning probability *ρ*^ℒ^ in GraphMorpher

**Fig 8:**
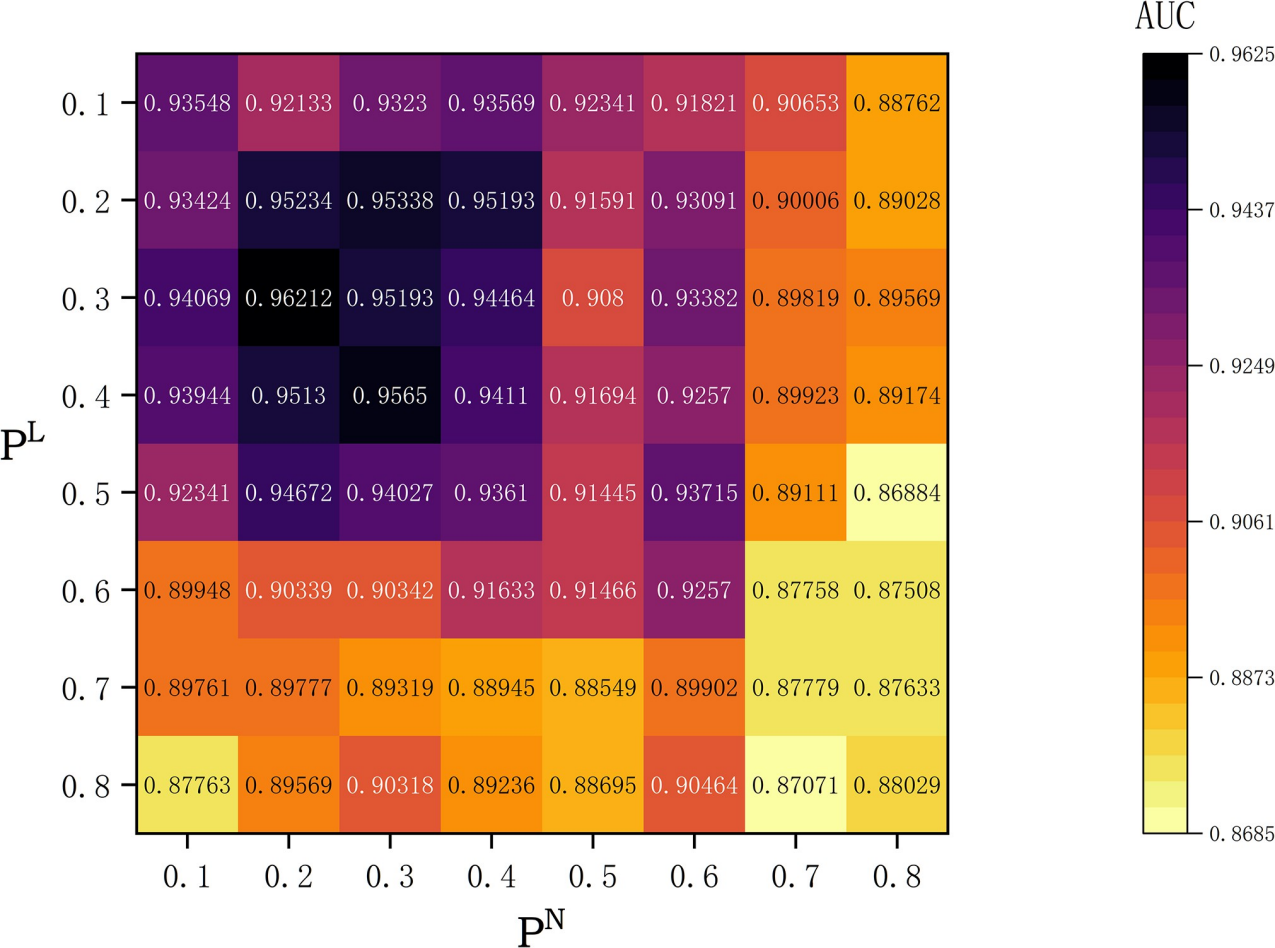
The parameter $\rho^{N}$ and *ρ*^ℒ^ impact the performance of the ASGCL model on the GDSC dataset. In the graph, the colors represent the magnitude of the ASGCL’s performance: darker colors correspond to higher AUC values, while lighter colors indicate lower AUC values.

As shown in Fig 8, the model exhibited different effects under the combined action of parameters $\rho^{N}$ and *ρ*^ℒ^. The experimental results indicate that the optimal choices for these two parameters are 0.2 and 0.3, respectively, under which the model achieved the highest AUC. Furthermore, Fig 8 demonstrates that an appropriate sparsity ratio can help the model remove redundant noise and enhance its learning ability, while an overly high sparsity ratio will damage the feature attributes and topological structure of the graph, reducing the model’s performance, thereby verifying the robustness and effectiveness of the GraphMorpher module.

## Conclusion

The main work of this article is to propose the ASGCL model, which utilizes contrastive learning and graph neural networks to extract features of cells and drugs and enhance the model’s discriminative ability for predicting drug resistance. This study conducted a large number of experiments, and compared with benchmark methods on actual datasets, the ASGCL model showed higher efficiency in predicting drug resistance tasks. In addition, the ASGCL model adopts an end-to-end training method to enrich the learned feature information. Overall, the experiment verified the reliability of the ASGCL model, which can accurately predict drug resistance tasks on sparse data. This indicates that end-to-end graph contrastive learning methods can help improve the predictive ability for drug resistance tasks. However, the ASGCL model still has some limitations. Future work will focus on finding more effective and universal graph data augmentation methods to further improve the performance and applicability of models.

## Discussion

Predicting cancer drug response is the main goal of using neural networks in the field of machine learning to assist cancer drug discovery and precision medicine in cancer research. In this work, we developed a model called ASGCL that utilizes multiple levels of contrastive learning tasks to enhance the model’s learning ability, providing a new deep learning framework for exploring the interactions between drugs and cell lines. The predictive ability of ASGCL has been validated on the GDSC dataset and compared with state-of-the-art methods under various evaluation settings. Extensive case studies have demonstrated the ability of ASGCL to interpret and predict cell lines and drug responses, as well as to search for relevant response outcomes.

Despite our efforts, there is still room for improvement. The performance of ASGCL in predicting missing drug and cell line CDRs was lower than expected. In such problems, due to the sparsity of the data type itself and the lack of prior knowledge of drugs/cell lines in the model, ASGCL can only fit the reaction process of missing data through existing data. Taking inspiration from prior knowledge in the medical field, introducing some regularization processes or knowledge graphs as prior knowledge into the model may greatly alleviate this problem. In addition, due to the inherent non interpretability of deep learning, it is expected that in future research, more mathematical methods or data refinement will be used to determine the role of models in predicting cell line drug interactions.

## Method

Now we introduce the ASGCL model and then describe its workflow in detail. The paper presents a method named the ASGCL model, aimed at predicting the degree of response between cell lines and drugs. The ASGCL model integrates linear and nonlinear features of cell lines with drug characteristics through utilizing nonlinear subspaces. It employs contrastive learning to extract efficient representations of interactions between cell lines and drugs, thereby predicting tasks related to drug resistance.

### Overview

**Fig 9:**
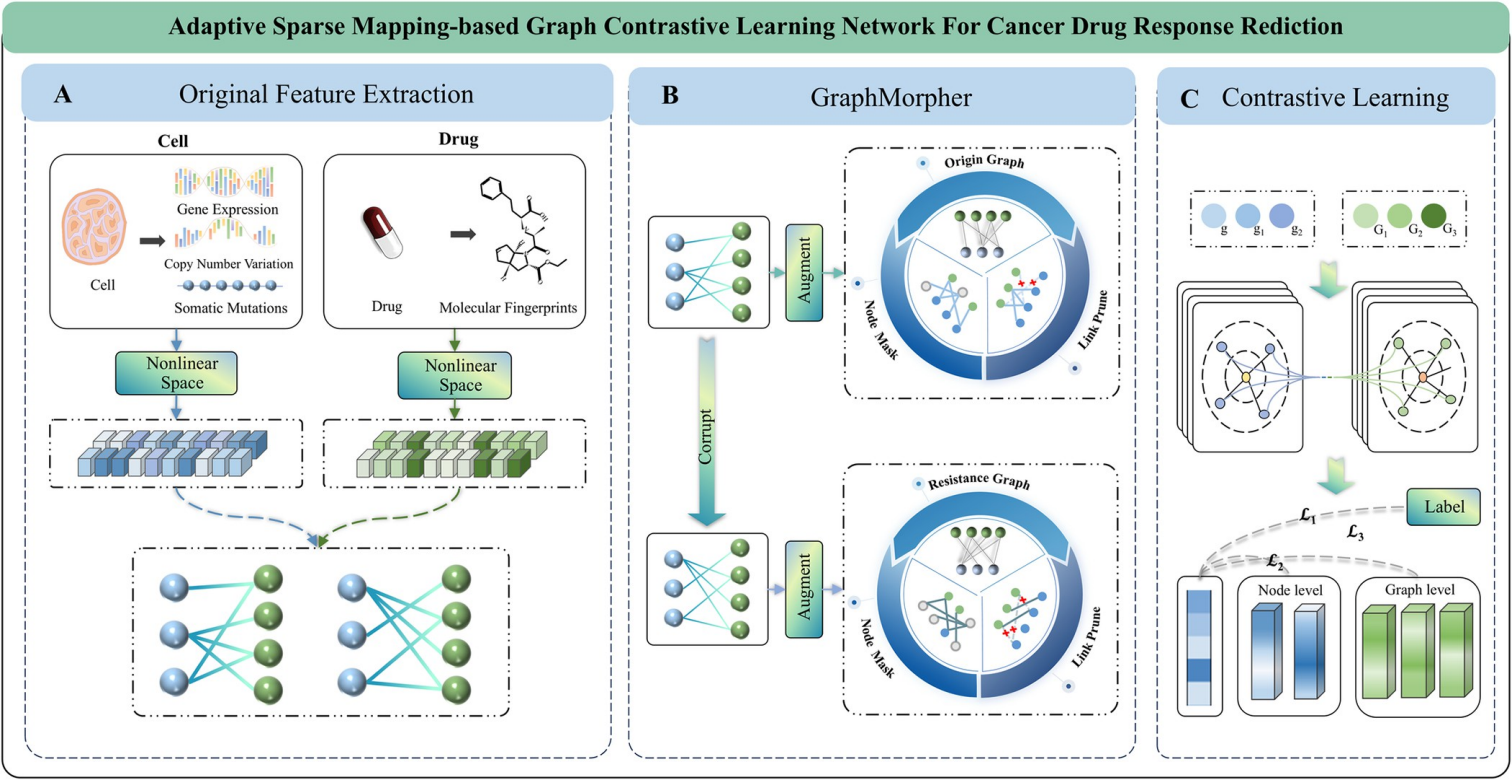
Schematic diagram of the ASGCL model. Module A utilizes a nonlinear subspace to extract cell line and drug features as primary characteristics; Module B, named GraphMorpher, adaptively sparsify the input graph structure; Module C is a contrastive learning module, which enhances the model’s discriminative ability by processing and comparing multiple graph structures.

The ASGCL model (Fig 9) initially constructs a nonlinear subspace to extract and integrate the linear and nonlinear features of cell lines and drugs, forming their primary characteristics. Subsequently, these features are mapped to various nodes of a heterogeneous graph, using the relationships between nodes to represent interactions between cell lines and drugs. Next, we design the GraphMorpher module to enhance the graph structure’s expressive capability, which transforms the original graph into multiple enhanced graphs by augmenting node attributes and topological structures within the graph. These enhanced graphs are then processed by a graph encoder, generating feature representations rich in semantic information. To further enhance the model’s feature expressiveness and generalization, we employ a contrastive learning strategy, defining positive and negative samples. This approach yields more discriminative feature representations through the comparison of positive and negative samples, thereby enhancing predictions for drug resistance tasks. Ultimately, the model optimization merges supervised loss with contrastive loss, where supervised loss guides the learning of task-specific knowledge, and contrastive loss facilitates the learning of diverse feature representations. Through the joint optimization of these two types of loss, a more accurate prediction of cell line and drug reactions is achieved.

### Nonlinear subspace

**Fig 10:**
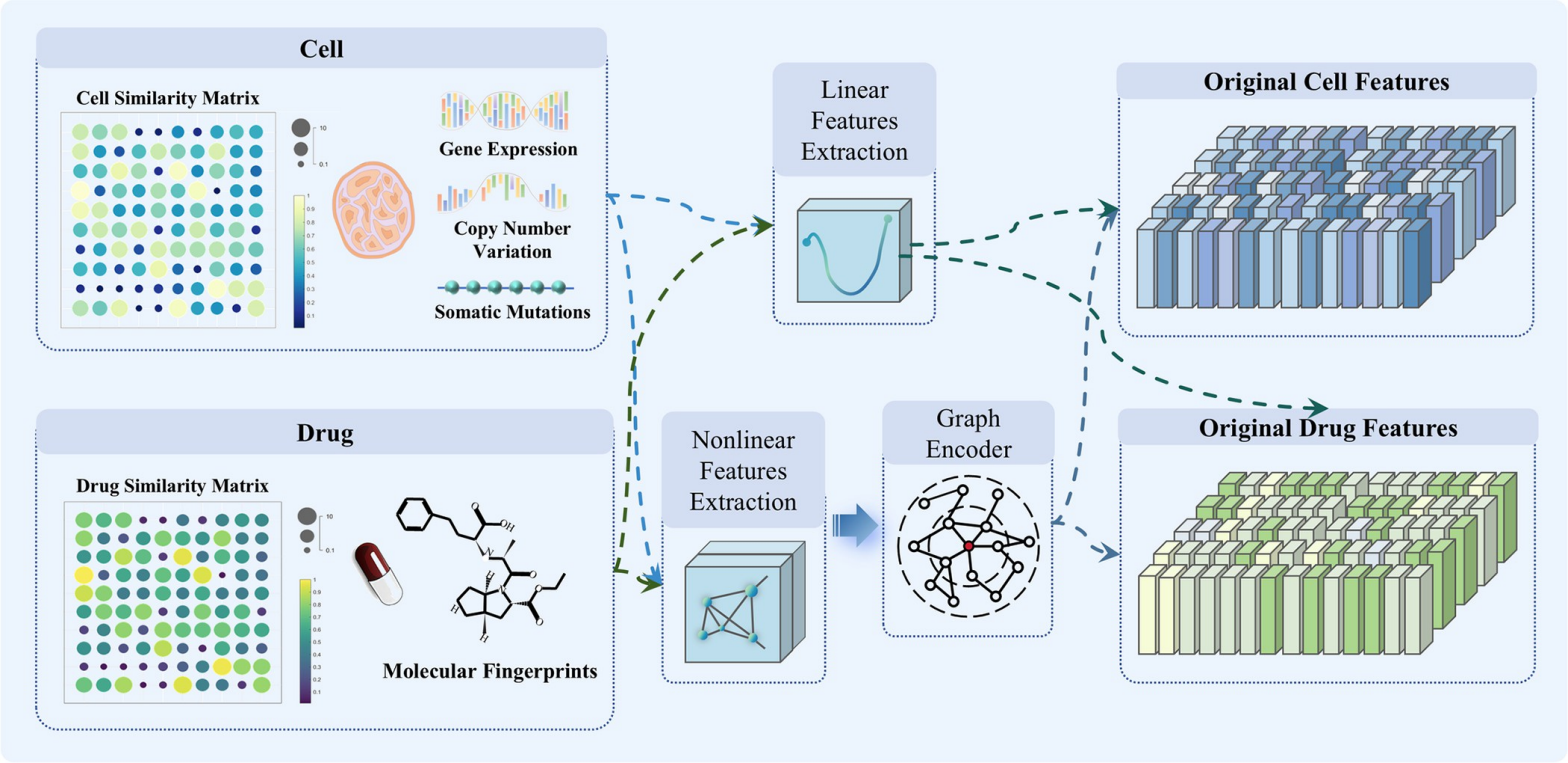
Schematic diagram of nonlinear subspace module.

This study aims to develop the ASGCL model to learn feature representations of cell lines and drugs, and to predict the degree of their interaction. To achieve this objective, the paper extracts features from two perspectives: linear relationships and nonlinear topological structures, as shown in Fig 10. Initially, linear features of cell lines and drugs are extracted by processing their multi-omics data through linear functions. Subsequently, the features of cell lines and drugs are mapped onto graph structures. Using graph encoders, the nonlinear topological structures of cell lines and drugs are extracted, learning the linkages between nodes in the graph to represent their nonlinear features. Ultimately, the linear and nonlinear features are integrated to form a comprehensive feature representation of cell lines and drugs. By combining linear and nonlinear features, this approach not only captures the complexity of cell line and drug data but also provides a more complete and rich feature representation. These features serve as inputs for the GraphMorpher module, aiming to enhance the accuracy of predictions for cell line-drug reactions.

### Linear features

The model inputs are multi-omics features of cell lines and molecular fingerprints of drugs. Matrix *A*∈*R*^*m*×*n*^ is used as the cell line-drug association matrix, where *m* denotes the number of cell lines and *n* the number of drugs. *A*_*ij*_ = 1 indicates that drug *j* is sensitive to cell line i; otherwise, *A*_*ij*_ = 0.

Since cell line gene expression and drug molecular fingerprint features usually have different dimensions, weight matrices *W*_*c*_ and *W*_*d*_ are used for linear transformation of cell line gene expression and drug expression, as shown in Eq 8:

$$E_{c}=C_{i}\times W_{c}$$ (8)

where *E*_*c*_ represents the characteristics of the cell line, and *C*_*i*_ represents the gene expression value of the i-th cell line in the gene expression matrix.

$$E_{d}=D_{i}\times W_{d}$$ (9)

where *E*_*d*_ represents the feature representation of the drug, and *D*_*i*_ denotes the expression value of the i-th drug in the drug molecular fingerprint matrix.

After obtaining the feature representations of cell lines and drugs, the features are normalized by calculating the normalization coefficients, as shown in Eq 10 and Eq 11:

$$N_{C}={\left(\sum_{j}A_{ij}+1\right)}^{-1}+I_{m}$$ (10)

$$N_{D}={\left(\sum_{j}A_{ji}+1\right)}^{-1}+I_{n}$$ (11)

where *N*_*C*_ and *N*_*D*_ respectively represent the normalized feature coefficients of cell lines and drugs, *I*_*m*_ is an m-dimensional identity matrix, and *I*_*n*_ is an n-dimensional identity matrix.

In summary, this paper employs linear functions to transform the original node features of cell lines and drugs into their linear features, as depicted in Eq 12 and Eq 13

$${LF}_{Cell}=\omega_{1}\cdot E_{c}\cdot N_{C}$$ (12)

$${LF}_{Drug}=\omega_{2}\cdot E_{D}\cdot N_{D}$$ (13)

where *ω*_1_,*ω*_2_ is a learnable weight parameter.

### Nonlinear features

This study assumes that similar drugs have similar reactions to similar cell lines. Considering network structure and feature attributes, the study maps cell lines and drugs onto an isomorphic graph, learning node similarities through a graph encoder to infer their reactions.

Cell lines are represented through a cell line similarity matrix, with the constructed graph *G*_*C*_ = (*X*_*c*_,*A*_*C*_) representing cell lines. In the graph *G*_*C*_, nodes represent cell lines and links between nodes indicate similarities among them. *X*_*c*_ is the feature representation of cell lines, recording the characteristics of all genes in the cell line; $A_{C}\in R^{N_{c}\times N_{c}}$ is the adjacency matrix of *G*_*C*_, indicating the degree of similarity among cell lines, where *N*_*c*_ is the number of cell lines.

Drugs are represented through a drug similarity matrix, with the constructed graph *G*_*D*_ = (*X*_*D*_,*A*_*D*_) representing drugs. In the graph *G*_*D*_, nodes represent drugs and links indicate similarities between them. *X*_*D*_ is the feature representation of drugs, recording the characteristics of all drugs; $A_{D}\in R^{N_{d}\times N_{d}}$ is the adjacency matrix of *G*_*D*_, indicating the similarity among drugs, where *N*_*d*_ is the total number of drugs.

Using the above methods, this study employs a graph neural network encoder to encode node features and their similarities in the graph, achieving effective propagation of feature information among related nodes. During this process, it captures the characteristics of cells and drugs and reveals the latent nonlinear feature representations in cell lines and drugs through the information propagation mechanism. This approach enables the study to more deeply understand and predict the complex interactions between cells and drugs.

After obtaining the linear and nonlinear features of cell lines and drugs, they are combined to obtain the final representation of cell lines and drugs, as shown in Eq 14 and Eq 15.


$$X_{C}={LF}_{Cell}+{NLF}_{Cell}$$ (14)

$$X_{D}={LF}_{Drug}+{NLF}_{Drug}$$ (15)

In summary, after integrating the linear and nonlinear features of cell lines/drugs through a nonlinear subspace, based on the sensitive response between cell lines and drugs, an undirected heterogeneous graph *G*_*Sen*_ = (*X*_*CD*_,*A*_*Sen*_) can be constructed. Here, $X_{CD}=\left[\begin{array}{c} X_{C}\\ X_{D} \end{array}\right]$ represents the sum of the cell line feature representation *X*_*C*_ and the drug feature representation *X*_*D*_; *A*_*Sen*_ is the adjacency matrix of *G*_*sen*_, representing the reaction relationship between cell lines and drugs,where *A*_*Sen*_ = 1 indicates sensitivity of the cell line to the drug, and *A*_*Sen*_ = 0 otherwise.

### GraphMorpher

**Fig 11:**
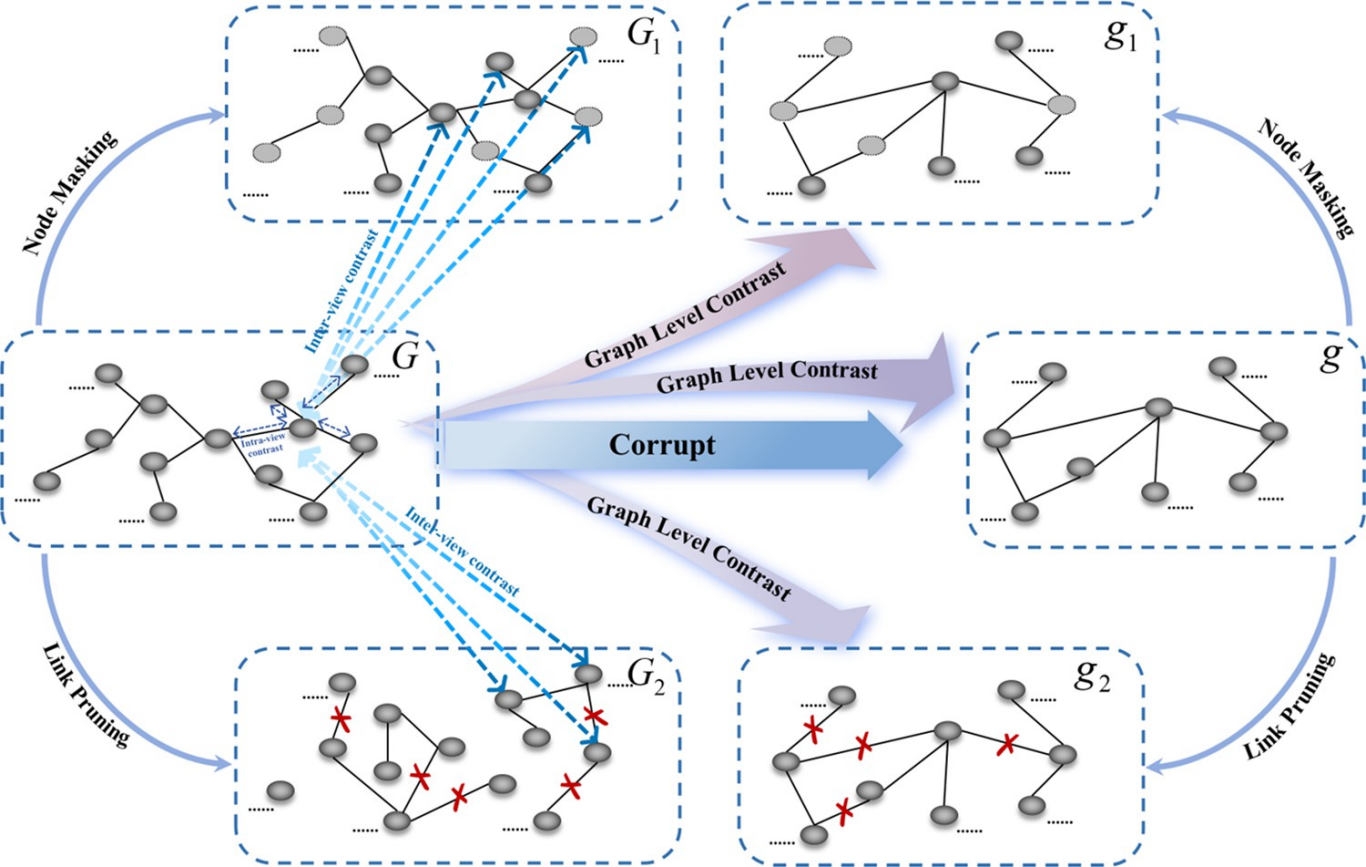
GraphMorpher diagram.

Based on the overall framework of contrastive learning [28,29,30,44,45,52], a GraphMorpher module is proposed. The primary function of GraphMorpher is to generate multiple enhanced views for the original graph *G*_*sen*_, which then aids in better feature extraction of *G*_*sen*_ within the contrastive learning module, as shown in Fig 11 and detailed in Algorithm 1:

**Algorithm 1:** GraphMorpher

**Input:** Original Graph:*G* = (X,A); Mask probability: $\rho^{N}$; Pruning probability:*ρ*^ℒ^

**Output:** $G;G^{N};G^{L};\tilde{G};{\tilde{G}}^{N};{\tilde{G}}^{L}$

1:  /**** Structure new heterogeneous graphs $\tilde{G}$

2:  Calculate $\tilde{A}$ via Eq 16

3:  $\tilde{G}\leftarrow (X,\tilde{A})$

4:  for *G* = (X,A) and $\tilde{G}=(X,\tilde{A})$ do

5:  /****Calculate sparsity probability

6:  Calculate $P_{Mask}$ via Eq 17 and Eq 18

7:  Calculate $P_{prune}$ via Eq 21 and Eq 22

8:  /**** Sampling masked nodes

9:  Calculate ***X***′ via Eq 20

10:  /**** Sampling pruned links

11:  Calculate ***A***′ via Eq 23

12:  /****Construct augment graph

13: ***G***^***N***^←(*X*′,*A*)

14: ***G***^***L***^←(*X*,*A*′)

15:  **end for**

16:  **Return** $G;G^{N};G^{L};\tilde{G};{\tilde{G}}^{N};{\tilde{G}}^{L}$

Inspired by the definition of positive and negative samples in contrastive learning, this paper inverts the adjacency matrix *A*_*Sen*_ of the original graph *G*_*sen*_ to obtain a new adjacency matrix *A*_*Res*_, as shown in Eq 16:

$$A_{ij}^{Res}=\{\begin{array}{c} 0,A_{ij}^{Sen}=1\\ 1,A_{ij}^{Sen}=0 \end{array}$$ (16)

The adjacency matrix *A*_*Sen*_ represents the sensitivity between cell lines and drugs; thus, the new adjacency matrix *A*_*Res*_ represents the resistance response between them. Keeping the node features *X*_*CD*_ unchanged and using the new adjacency matrix *A*_*Res*_, a new resistance graph *G*_*Res*_ = (*X*_*CD*_,*A*_*Res*_) is formed.

It is believed that using completely opposite links to construct graph *G*_*Res*_ allows *G*_*sen*_ to improve its learned features by maximizing the distance to *G*_*Res*_, thereby enhancing the model’s discriminative ability.

Upon obtaining graph *G*_*Res*_, an adaptive graph augmentation method was designed based on node attributes and topological structure in the graph. Specifically, for any given graph structure, two new views are generated by adaptively masking original node attributes and pruning the topological structure; then, contrastive loss is computed to maximize the consistency of node representations in these two views; finally, through contrastive learning between nodes, the final node representation is obtained for predicting the degree of reaction between cell lines and drugs. This method enables the model to better learn node features, forcing it to recognize the semantic information of nodes; moreover, it prunes links based on the graph’s topological structure, thereby highlighting important link structures.

### Node mask

At the node attribute level, this study adopts an adaptive approach to mask the attributes of nodes in the graph, by masking nodes with sparse information and emphasizing nodes with rich information, thus better extracting underlying semantic information. In contrast, many previous studies [52,53] have destructively pruned nodes, damaging not only the underlying semantic features of the nodes but also affecting the topological structure between connected nodes. Particularly in drug resistance prediction tasks, due to the inherent sparsity of medical data, such destructive methods further sparsify the data, preventing the extraction of key features.

In the process of calculating node masks, considering the amount of information contained in nodes, F(v) is defined as the centrality of the feature vector of node *v* [54,55]. F(v) takes into account the importance of neighboring nodes, thereby assigning different contributions to each neighbor. F(v) is calculated as the eigenvector corresponding to the largest eigenvalue of its adjacency matrix, representing the most contributing factor. The F(v) of each node is proportional to the sum of F(v) of its neighboring nodes, and nodes connected to many neighbors or to nodes with greater influence will have a higher F(v). In this paper, features that frequently appear in nodes are considered important, thus a centrality measure $M_{i}^{N}$ is defined for each node v, and $M_{i}^{N}$ is used as the measure of node *v*′s features, as shown in Eq 17:

$$M_{i}^{N}=\sum_{v\in V}{|x}_{v}^{i}|\cdot F(v)$$ (17)

where ${|x}_{v}^{i}|$ representing the feature size of node *v* in dimension F(v) represents the contribution of the node. Next, the probability $P_{Mask}$ of a node being masked is obtained through normalization using weights, where $P_{Mask}$ indicates the importance of the node, as shown in Eq 18.

$$P_{Mask}=\min\left(\rho^{N}\cdot \frac{M_{max}^{N}-M_{i}^{N}}{M_{max}^{N}-\mu_{M}^{N}},T_{N}\right)$$ (18)

where $\rho^{N}$ controls the probability of node masking, $M_{max}^{N}$ and $\mu_{M}^{N}$ are the maximum and average values of $M_{i}^{N}$, respectively, and $T_{N}$ is the truncation probability of node masking, used to prevent excessively high masking probabilities, as overly high probabilities would lead to excessive sparsity of semantic features in the graph, making it impossible to extract effective semantic features in subsequent encoding processes.

After obtaining the masking probability $P_{Mask}$ of nodes, a portion of node dimensions and some feature information are adaptively masked according to $P_{Mask}$.

In the adaptive masking phase, sampling is first performed on a random vector *m*∈{0,1}, where each dimension of m is independently sampled according to $P_{Mask}$ from a Bernoulli distribution, as shown in Eq 19.


$$m_{i}∼Bern(1-P_{Mask})$$ (19)

Subsequently, the generated node feature X is shown in Eq 20:

$$X=x_{1}\cdot m_{i};x_{2}\cdot m_{i};\ldots \ldots \ldots x_{N}\cdot m_{i}$$ (20)

where operators (;) and (………) represent connection operators, and (∙) represents element multiplication operations.

### Link pruning

In processing the graph structure, this study employs an adaptive pruning method to enhance the graph structure. By setting different pruning probabilities, the graph structure is refined, removing links with less information and emphasizing key links, thereby optimizing the information density and analysis process of the graph, focusing more on key features. This approach not only enhances the information density of the graph structure but also optimizes the subsequent graph analysis process, enabling more effective capture and utilization of key information in the graph, thus significantly improving the predictive performance of the model.

For each link, this paper defines a link centrality measure $M_{ij}^{L}.\;M_{ij}^{L}$ is based on the node centrality measures $M_{i}^{N}$ and $M_{j}^{N}$ of the nodes at both ends of the link (*i*,*j*), defining its degree of influence. A logarithmic function is applied to mitigate the impact of nodes with highly dense links, as shown in Eq 21:

$$M_{ij}^{L}=\log(\frac{M_{i}^{N}+M_{j}^{N}}{2})$$ (21)

The probability of pruning links in the graph, $P_{prune}$, is adaptively adjusted based on the $M_{ij}^{L}$ of each edge, thus maintaining the integrity of the graph’s topological structure, as shown in Eq 22:

$$P_{prune}=\min\left(\rho^{L}\cdot \frac{M_{max}^{L}-M_{ij}^{L}}{M_{max}^{L}-\mu_{M}^{L}},T_{L}\right)$$ (22)

where *ρ*^ℒ^ controls the probability of link pruning, $M_{max}^{L}$ and $\mu_{M}^{L}$ are the maximum and mean values of $M_{ij}^{L}$, respectively, and $T_{L}$ is the truncation probability of the link, used to prevent excessively high probabilities of pruning, ensuring that the graph structure does not suffer from destructive information loss. Formally, based on the pruning probability $P_{prune}$, pruning operations are performed in the original set of links ℒ, sampling a pruned subset ℒ_*sub*_, as shown in Eq 23:

$$P\{((i.j)\in L_{sub}\}=1-P_{prune}$$ (23)

where *P*{((*i*.*j*)∈ℒ_*sub*_} is the probability that the link between nodes i and j is retained. The subset ℒ_*sub*_ represents the collection of remaining links after pruning the original set ℒ.*P*_*prune*_ is the pruning probability for edges, reflecting the importance of links. The more important the link, the lower the probability it will be pruned. This pruning operation is more likely to prune unimportant links, while keeping important link structures intact in the enhanced view.

By using the aforementioned adaptive pruning method, the input graph structure can be sparsified, focusing on reducing graph structures with less information while preserving key topological structures in the graph, thereby highlighting important structural features in the graph. In contrastive learning, the adaptively sparsified graph structure helps the original graph to learn and reinforce key topological structure features more efficiently. This strategy not only improves the efficiency and accuracy of feature extraction but also provides a more solid foundation for deeply understanding and predicting complex network behaviors.

Finally, through the proposed node masking and link pruning operations, this paper generates two updated views based on the node features and topological structure of the input graph. In experiments, different masking probabilities $\rho^{N}$ and pruning probabilities *ρ*^ℒ^ are set to provide the graph encoder with diverse contextual semantic features. The GraphMorpher module randomly and adaptively generates enhanced graphs in each iteration, maintaining important structures and attributes while perturbing potentially unimportant features and topological structures. In this paper, the graph generated from *G*_*sen*_ through node masking is denoted as $G_{Sen}^{N}=(X_{CD}^{M},A_{Sen})$, and the graph generated through link pruning is denoted as $G_{Sen}^{L}=(X_{CD},A_{Sen}^{P})$; similarly, *G*_*Res*_ generates $G_{Res}^{N}=(X_{CD}^{M},A_{Res})$ and $G_{Res}^{L}=(X_{CD},A_{Res}^{P})$.

### Contrastive learning

This study focuses on contrasting node attributes and link structures within the graph structure, utilizing positive and negative sample analysis to learn feature representations, constructing a multi-relational link model for drug resistance prediction, and employing a graph encoder to extract features of cell lines and drugs. In the graph structure model, neighbor nodes of cell lines or drugs propagate different information in various interaction environments. Therefore, in determining the network structure, different information propagation paths were considered, including various types of encoders and the scope of K-order neighborhoods. By effectively utilizing information sharing among nodes in the graph and aggregation operations in neighborhoods, the information of neighbor nodes is aggregated, thus obtaining the final node embeddings. Referring to the definition of positive and negative samples in contrastive learning, samples of different levels were set up in the experiments. At the node level, node-level contrastive learning is conducted by comparing the original graph and the graph processed by GraphMorpher; similarly, at the graph level, graph-level contrastive learning is conducted by comparing the original graph and the resistance graph processed by GraphMorpher. In node-level contrastive learning, positive and negative samples for each node of a given graph are defined, where the same nodes in other graph structures are considered as positive samples, and different nodes as negative samples. In the graph-level contrast task, inter-sample learning is based on node-level and graph-level features. In the contrastive learning process, based on the defined node-level and graph-level positive and negative samples, the model strives to minimize the distance between positive samples while maximizing the distance between negative samples, thereby ensuring the consistency of encoded embeddings of each positive sample and effectively distinguishing the features of negative samples. Therefore, by analyzing the feature representations of the original graph through the contrastive learning process from multiple views, these features can be used for subsequent predictions of cell line-drug reactions.

### Node level contrastive learning

In the node-level contrastive learning task, for node *v*_*i*_ in any view *v*, only node *u*_*i*_ in the generated view u is its corresponding positive sample, and these two nodes are considered as the current positive sample pair, while other nodes in these two views are considered as negative samples. For each node-level positive sample pair, this paper considers that negative samples have two different sources: intra-graph negative samples and inter-graph negative samples.

In the node-level contrastive learning task, this paper defines a discriminator *θ*(*u*_*i*_,*v*_*i*_) = *s*(*g*(*u*_*i*_),*g*(*v*_*i*_)), where *s*() represents cosine similarity, and *g*() represents a two-layer MLP nonlinear projection function, thus enhancing the discriminative power of the discriminator. For node-level positive sample pairs, the distance between positive sample pairs is defined as $C_{pos}=e^{\theta (u_{i},v_{i})/\tau }$, where an exponential function is used to amplify the similarity between positive samples, and τ is the temperature parameter. Similarly, the distance between intra-graph positive and negative sample pairs is defined as $C_{neg}^{intra}=e^{\theta (u_{i},u_{k})/\tau }$, and the distance between inter-graph positive and negative sample pairs as $C_{neg}^{inter}=e^{\theta (u_{i},v_{k})/\tau }$.

Based on the above definition, for each node level sample pair (*u*_*i*_,*v*_*i*_), the learning objectives of the node level samples are defined as:

$$h(u_{i},v_{i})=\log\frac{C_{pos}}{C_{pos}+\sum_{k=1}^{N}C_{neg}^{intra}+\sum_{k=1}^{N}C_{neg}^{inter}}$$ (24)

The loss of other positive sample graph structures is defined the same as the above loss. In order to ensure the maximization of the overall goal, this article defines the goal of average graph level positive sample pairs as:

$${Loss}_{nod}=\frac{1}{2N}\sum_{i=1}^{N}\left(h(u_{i},v_{i})+h(v_{i},u_{i})\right)$$ (25)

Defining node-level positive and negative samples in contrastive learning tasks enables the model to maximize its learning of effective feature representations from node-level positive samples, thereby enhancing the extraction of effective features from the original graph.

### Graph level contrastive learning

In the graph-level contrastive learning task, this study contrasts the features of the original graph *G*_*sen*_ with its resistance graph *G*_*Res*_ and their generated graphs, using both node-level and graph-level features to enhance the model’s generalization ability. Similar to the node-level contrastive learning task, in the graph-level task, for any node *v*_*i*_ in a given view, only the graph-level feature *V* of *v*_*i*_’s view *v* is its positive sample, while the graph-level features of other graph structures serve as negative samples for node *v*_*i*_. This paper posits that the completely opposite topological structure in the resistance graph *G*_*Res*_ compared to the original graph *G*_*sen*_ represents the antagonistic response between cell lines and drugs. Using two distinctly different topological structures for contrastive learning can enhance the model’s discriminative ability.

In the graph-level contrastive learning task, another discriminator *φ*(*v*_*i*_,*V*) = *σ*(*v*_*i*_^*T*^ *W*_*V*_) is defined, where *σ*() represents a sigmoid non-linear function, *W*_*V*_ is a learnable scoring matrix, and *φ*(), constructed by *σ*(), serves as a contrast discriminator to estimate the similarity between node-level embeddings and graph-level embeddings.

For the graph level, this study aims to maximizes the divergence between node-level features and graph-level features to achieve the objective of contrastive learning, which is defined as:

$$f(v_{i},u_{i},V)=\sum_{i=1}^{N}\log(\varphi (v_{i},V))+\sum_{i=1}^{N}\log\left(1-\varphi (u_{i},V\right))$$ (26)

The loss for the rest of the graph structures is defined similarly to the above. To ensure the maximization of the overall objective, the paper defines the objective for average graph-level sample pairs as:

$${Loss}_{gra}=\frac{1}{2N}\sum_{i=1}^{N}\left(f(v_{i},u_{i},V)+f(u_{i},v_{i},U)\right)$$ (27)

where *u*_*i*_ is the corresponding node feature in the generated graph to node *v*_*i*_, and *U* is the graph-level feature. Estimating node-level features and graph-level features among graph-level negative samples can enable the model to learn graph-level features to the maximum extent.

In summary, in each training round, cell line and drug data are input into a nonlinear subspace to merge linear and nonlinear features; and based on the heterogeneity of cell lines and drugs, a heterogeneous graph is constructed and input into the GraphMorpher module for graph enhancement, followed by a graph reasoning process using a graph encoder to obtain node representations of the graph; using the graph encoder for inference of node representations, the original graph features are finally optimized through contrastive learning for drug resistance prediction tasks.

For the final prediction of drug resistance tasks, the final embeddings of cell line nodes and drug nodes are utilized, and the probability of their sensitive response is predicted using a scoring function with an inner product.
